# Development and preliminary validation of a questionnaire to measure satisfaction with home care in Greece: an exploratory factor analysis of polychoric correlations

**DOI:** 10.1186/1472-6963-10-189

**Published:** 2010-07-05

**Authors:** Vassilis H Aletras, Arsenis Kostarelis, Maria Tsitouridou, Dimitris Niakas, Anna Nicolaou

**Affiliations:** 1 Department of Business Administration, University of Macedonia, 156 Egnatia str., P.O. Box 1591, Thessaloniki 54006, Macedonia, Greece; 2 Faculty of Social Sciences, Hellenic Open University, Patra, Greece

## Abstract

**Background:**

The primary aim of this study was to develop and psychometrically test a Greek-language instrument for measuring satisfaction with home care. The first empirical evidence about the level of satisfaction with these services in Greece is also provided.

**Methods:**

The questionnaire resulted from literature search, on-site observation and cognitive interviews. It was applied in 2006 to a sample of 201 enrollees of five home care programs in the city of Thessaloniki and contains 31 items that measure satisfaction with individual service attributes and are expressed on a 5-point Likert scale. The latter has been usually considered in practice as an interval scale, although it is in principle ordinal. We thus treated the variable as an ordinal one, but also employed the traditional approach in order to compare the findings. Our analysis was therefore based on ordinal measures such as the polychoric correlation, Kendall's Tau b coefficient and ordinal Cronbach's alpha. Exploratory factor analysis was followed by an assessment of internal consistency reliability, test-retest reliability, construct validity and sensitivity.

**Results:**

Analyses with ordinal and interval scale measures produced in essence very similar results and identified four multi-item scales. Three of these were found to be reliable and valid: socioeconomic change, staff skills and attitudes and service appropriateness. A fourth dimension -service planning- had lower internal consistency reliability and yet very satisfactory test-retest reliability, construct validity and floor and ceiling effects. The global satisfaction scale created was also quite reliable. Overall, participants were satisfied -yet not very satisfied- with home care services. More room for improvement seems to exist for the socio-economic and planning aspects of care and less for staff skills and attitudes and appropriateness of provided services.

**Conclusions:**

The methods developed seem to be a promising tool for the measurement of home care satisfaction in Greece.

## Background

The elderly in the European Union constitute a significant segment of the society, which is expected to rise in the future [1]. In 2005 there were 79 million individuals over the age of 65 in Western and Central Europe. This figure will increase to 107 million in 2025 (+ 35%) and to 133 million in 2050 (+ 68%). The largest growth in this age group will be observed for those over 80 years (+ 180%). By 2030, in contrast, all other adult age groups will be declining in size. There is therefore a gradual shift from a society dominated by young individuals to a society in which the elderly will become the majority. The need for the provision of social services is exacerbated by the observed social exclusion, financial difficulties and health problems related to this particular age group. The World Health Organization has thus placed "good aging" fifth among its targets for the 21^st ^century.

This has led to the adoption of practices which aimed at the promotion of the well-being of the elderly on a social as well as on a physical level. Home care programs have been evolving in European countries for many decades based on the aforementioned and comprise the most important attempt to support the vulnerable social group of the elderly [2]. As one of the few social initiatives of local government in Greece ten years ago, legislation was enacted in the majority of municipalities for the elderly and people with disabilities. The program aimed at improving the quality of life of the enrollees. Its effectiveness nevertheless has never been formally assessed. This is not surprising since research on patient satisfaction by means of validated questionnaires is in fact very scarce in Greece [3,4].

The program of providing home care services to the elderly and disabled begun in 1996 and quickly spread to most municipalities in Greece [5]. Seventy five percent of the necessary funds have been provided by the European Union and the rest through national financing [6]. By the beginning of 2005 there were 1084 home care units established serving 40,060 enrollees [7]. The general purpose of the program was to confront the social exclusion and institutionalization as well as to improve the quality of life of the elderly and the disabled. Priority has been given to individuals with an inability for self-care, who lived alone and had limited financial resources to improve their living standards and become independent.

In order to achieve these goals, social work, nursing care and life assistance services have been used [8]. The social worker is responsible, at an initial stage, for the needs assessment of the applicants, the scheduling of home visits and the determination of the range of provided services. Moreover, this employee is assigned with the task of co-operating with local parties, welfare services, voluntary organizations and the linking of the home care program with the participants and the local community. His/her role also includes provision of counseling and emotional support to the enrollees and their caregivers as well as admission to various institutions (e.g. hospitals, welfare, mental health units) when necessary. Nursing care is prevention-oriented. Information is provided regarding the illnesses and diseases enrollees are facing or are likely to face in the future. Nursing also addresses the enrollees' nutrition. Blood tests (including blood glucose tests), examination of vital signs and medication compliance are targeted towards the preservation of health, the self-care of enrollees and the establishment of a safe environment. Information is provided and the family and other caregivers are trained, so that they can handle problems more effectively. The aide workers' assistance includes help with personal hygiene, light housework, grocery shopping or other small purchases, payment of bills, meal preparation and other life-assisting activities. The interaction of all team members is often essential, especially for activities such as companionship, social mobilization of enrollees, emotional and psychological support and co-operation with caregivers.

In this study we developed and tested a questionnaire for measuring home care satisfaction in Greece. Although other questionnaires were available on an international level, there are significant differences in the various national settings that made the development of a customized instrument necessary. First, in several countries the specific home care services enjoyed by the participants depend on private payments or a person's contribution to a health insurance fund, whereas in Greece all services are provided free of charge by the municipalities. They are therefore determined solely by assessing relative needs. Second, in Greece the team which offers the home care services comprises mainly of a social worker, a nurse and two aide workers, whereas other specialties (medical doctors, psychologists, physiotherapists) encountered in other countries might or might not be included, depending on the specific municipality's policy. In addition, other services, such as meal preparation, might be available to all upon request abroad, yet only in certain programs and individuals in Greece. In general, there are important differences in the structure and mode of provision of home care services between countries, related to the methods of financing, as well as the cultural and social factors.

The aim of this study was to measure home care satisfaction by developing an appropriate questionnaire that covers the Greek home care provision. The tool devised was psychometrically tested and subsequently the level of satisfaction with home care services was measured. A novel concept in this work is the use of polychoric correlations in the factorial analysis and psychometric testing, which acknowledges the ordinal nature of the data, typically ignored in relevant prior research.

## Methods

A questionnaire in Greek was developed to measure the satisfaction of enrollees with home care services. Items were phrased using a typical 5-point Likert scale with 1 indicating "strongly disagree" and 5 corresponding to "strongly agree". Positively and negatively worded items were interchanged to avoid positive biases [9]. Negative worded questions were therefore subsequently re-coded so that higher values would indicate higher levels of enrollee satisfaction, and *vice versa*.

Items were carefully selected after an extensive literature search and cognitive interviews with 15 program enrollees (of various ages, levels of education and incomes) and 15 employees (an equal number of social workers, nurses and aide workers) that were not included in the main study. Items were phrased so as to avoid vagueness and biases, double negatives, double-barreled questions and protests.

Satisfaction items reflect all the important aspects of the home care program, namely effectiveness, safety, respect, attention, participation in planning, correctness of planning, access, socialization and technical abilities of staff. The literature sought included theoretical papers, questionnaire validations and qualitative analyses [10-29]. Two questions included referred to general satisfaction from the provided home care services. Regarding socioeconomic and demographic variables, we recorded gender, age, educational level, income, family status, enrolment duration, visit frequency and informal caregivers [10]. Two more items were incorporated in order to exclude individuals that gave inconsistent responses. These had the same meaning as other questions but were differently phrased.

An important subsequent step in instrument development was pretesting, which took the form of cognitive interviews with elderly and disabled participating in the programs [30]. There were 10 such subjects from each group. These identified problems with the content and understanding of the questions, as well as additional causes of (dis)satisfaction, not captured by the instrument, thus enhancing reliability and content validity. Towards the same end, individuals pinpointed questions identified by the literature search that had to be removed from the instrument as being unimportant. The same persons were approached after a revision of the questionnaire and no additional issues were identified. Moreover, expert panel judgment was also exploited to ensure content validity and item clarity.

We initially screened the data by assessing (Spearman's) correlation coefficients between the items capturing satisfaction from specific characteristics of home care and the two items measuring overall satisfaction. Theory suggests that there might be a relationship apparent between overall satisfaction and satisfaction from individual services [9,31]. Specifically, overall satisfaction emerges from customer's evaluations of service encounters, which in turn are evaluated on the basis of service attributes (e.g. response time of the nurse). The latter are evaluated on the basis of positive and negative events that occur during the encounter with the provider. In this sense, the overall evaluation of the provider might be a weighted average of all evaluations of service attributes, the weights being the importance assigned by the customer to each attribute.

In our context, however, one needs additional justification for excluding items from the survey, than simply observing insignificant correlations, for two reasons. First, we employed in this research two questions that have been used most frequently in practice for measuring overall satisfaction. It is nevertheless theoretically possible that they do not fully cover all the aspects of satisfaction from the service encounter, as perceived by customers. Second, a single item might have to move a great deal in order to have a significant effect on overall quality and there might be additional effects of the particular item when combined with other related questions. For these reasons, statistically insignificant correlations observed in this study only served as triggers for further investigation of specific items.

The questionnaire was administered to enrollees of home care programs, who were elderly and disabled in the age group of 65 to 90 years. An inclusion criterion to the study entailed that the subjects should have been participating in the program for at least two months, following Westra *et al*. [15]. The sample was approached from March 7 to April 3, 2006 with face-to-face interviews by trained interviewers. The study was approved by the Review Board of the Hellenic Open University. Subjects were told that their anonymity would be assured in the study. We excluded those who faced health disorders/problems that affected their mental state (e.g. Alzheimer's disease, severe stroke). Subsequently, five home care programs in Thessaloniki were randomly selected from a total of 36 programs that existed in municipalities of the city having more than 10,000 residents: Municipality of Stavroupoli (two home care programs), Municipality of Thermaikos, Municipality of Menemeni and Pefka Village. Although a consensus is lacking, samples with at least 200 persons and an observation-to-item ratio higher than 5:1 are generally acceptable in this type of research [32]. The interviewers visited the houses of all 319 enrollees in these programs that matched the inclusion criteria at the time of the study.

Summated scales were formed by exploratory factor analysis [33]. The Kaiser-Meyer-Olkin measure of sampling adequacy and Bartlett's test of sphericity were used to check whether the data were suitable for factor analysis. Values of KMO greater than 0.70 have been characterized as "middling" (and greater than 0.90 as "marvelous"). Bartlett's test of sphericity tests the null hypothesis that the correlation matrix is an identity matrix (i.e. that there is no relationship between the items) [34].

Although in many instances they yield similar results, factor analysis was preferred to principal components analysis, since the purpose of the study was to develop a theoretical construct, rather than merely perform data reduction [35]. The specific factor analyses employed unweighted least squares and varimax rotation. The unweighted least squares method was chosen because it is robust, can be used with small samples and also when the correlation matrix is non-positive definite. Factors whose eigenvalues exceeded unity were retained [36]. Following other researchers, we assigned an item to a factor - summated scale if its factor loading was greater than 0.50 and its differences with other factor loadings of the same item were greater than 0.20 [37,38].

The main factor analysis carried out in this study acknowledges the fact that the 5-point Likert scale is in fact ordinal [39]. It was hence based on polychoric correlations. The polychoric correlation coefficient assumes that pairs of ordinal scores are in fact generated by latent bivariate normally distributed random variables. Measuring the association between ordinal variables entails the estimation of the product moment correlation between the corresponding normally distributed variables. Interestingly, a recent study that utilized a generalized polychoric coefficient found that it does not outperform the typical polychoric measure in cases where the sample size is smaller than 400 observations and the number of ordinal categories in the response scale is greater than three [40]. The polychoric correlation matrix was estimated using the two-stage procedure described in Jöreskog [41] and implemented in PRELIS v.2.8 [42]. Note that since the matrix was estimated in a pairwise fashion it was possible to be non-positive definite. An exploratory factor analysis was then performed entering the estimated polychoric correlation matrix into SPSS v.15.

Since prior research has mainly assumed that the Likert scale can be treated as an interval or ratio scale, we also performed, for comparative purposes, typical factor analyses based on Pearson correlations. In addition to unweighted least squares, we also extracted factors with principal axis factoring to account for the skewness observed in the data [43,44].

Internal consistency reliability measures the extent to which all items within a scale are indeed capturing the same construct. Cronbach's alpha coefficients greater than 0.80 indicate high levels of internal consistency, whereas values less than 0.70 suggest that the researcher should attempt deleting individual items from the scales to examine whether consistency improves [45]. Due to the ordinal nature of the data, an ordinal standardised item alpha coefficient was also derived by inserting polychoric correlations into the simplified formula of the alpha coefficient [46,47]. Note that prior research has examined the potential effect of skewness to this form of reliability coefficients and has found no impact of square root and log transformations on Cronbach's alpha coefficients [48]. A further criterion used to assess internal consistency reliability was inter-item correlations. Prior research has argued that items within a scale with lower correlations than 0.30 might not be sufficiently related to be used as measures of the same phenomenon, whereas items with correlations higher than 0.70 might be redundant [49]. We calculated Spearman's coefficients, thus taking into account potential skewness.

The degree to which scales are sensitive to external factors in successive measurements was assessed by examining test-retest reliability. The second administration occurred two weeks after the initial measurement. In this study, intraclass correlations were preferred to Pearson product moments coefficients because the test and retest variables originated from the same group of individuals and the former coefficients could detect systematic errors [50,51]. There is no consensus regarding the appropriate standards for the value of the coefficients [52]. Nevertheless, a value of 0.70 is considered satisfactory in practice [53]. In addition, to account for possible skewness in the data, we also employed Kendall's Tau b, which is a distribution-free correlation measure.

Multi-trait analysis was then performed to assess internal consistency reliability, construct validity and within-scale item additivity. It is a psychometric technique applied when a questionnaire is hypothesized to capture several distinct theoretical constructs - concepts, each measured by several individual items (questions). The criteria assessed are [54]:

• Each item should have a high correlation with all other items in the summated scale in which it is assumed to belong (item internal consistency). 0.40 represents the lowest acceptable correlation reflecting consistency.

• The correlation of an item with the sum of all other items in its scale should be higher than its respective correlations with all other summated scale scores (item discriminant validity).

• For items within a summated scale, the correlation between one item and the sum of the other items should be of roughly the same magnitude for all items (equal item-own scale correlation).

• Items in the same summated scale should have roughly equal variances.

If criteria 1 and 2 are not met, the groupings should be reconsidered and if criteria 3 and 4 are not met, weighting of items while forming summated scales should be pursued.

In the multi-trait analysis conducted we acknowledged the ordinal nature of the data as well as the potential skewness of individual item scores. We therefore compared Pearson, Spearman, polychoric and polyserial correlation results, following prior research [55]. In the first case, we used a t-statistic for testing the significance of the difference between two dependent correlations [56]. Spearman's Rho is suitable for ordinal data since it is based on ranks of data. Note also that wherever a variable (multi-item scale) had more than 15 categories, it was treated as continuous. Therefore, a polyserial correlation was used for its association with other ordinal variables.

Construct validity was further assessed by examining inter-scale correlations. These had to be statistically significant and greater than 0.40 [57]. Furthermore, we looked at the correlations of the global satisfaction measure with the other scales, as well as the correlations of the latter with specific participant characteristics. To account for data ordinality and skewness we computed polychoric, polyserial, Spearman and Kendall's Tau b coefficients.

Ceiling and floor effects for the created multi-item scales were examined by assessing the percentage of responses falling into the highest and lowest possible satisfaction scores (i.e. strongly agree and strongly disagree). These are measures of the sensitivity of the tool. Finally, we computed the means and medians of the created scales in order to measure satisfaction with various aspects of home care as well as overall enrollee satisfaction. In fact, we linearly converted the scores into a 0-100 point scale.

## Results

Initially, the questionnaire created included 33 items measuring satisfaction with individual aspects of home care. For most items, Spearman correlation coefficients between items and the two questions of overall satisfaction were statistically different from zero (p < 0.05). In contrast, two questions meant to capture satisfaction with individual attributes of home care had correlations with overall measures that were insignificant. These were "I believe there should be more staff (e.g. nurses, social workers) involved in the program" and "The home care program is in need of additional specialties (e.g. physicians, physiotherapists)". The reason why we eventually decided to exclude these two items was that they seem to refer to somehow technical aspects of care that the enrollees might not be to an extent able to answer correctly. Content validity does not seem to be sacrificed from this exclusion due to the existence of items 11 and 13, which ask enrollees whether they believe they should be visited more frequently and whether their needs are met. The items measuring individual aspects of enrollee satisfaction we used in the statistical analyses were therefore 31. Two more questions reflected overall satisfaction with home care programs. These are shown in Table 1 (Additional file 1 contains the original questionnaire).

**Table 1:** Description of items measuring satisfaction with home care

Item	Description	Mean	Median	Skewness Statistic	Standard Error
1	Help of staff in overcoming personal problems of the enrollee	4.16	4.00	-1.645	0.172
2	Encouragement in taking initiatives	3.75	4.00	-0.792	0.172
3	Staff disparages enrollee due to his/her personal problems	4.49	5.00	-2.285	0.172
4	Improvement in financial condition due to the provided services	3.86	4.00	-0.803	0.172
5	Staff is in a hurry to leave the house	4.10	4.00	-1.341	0.172
6	Staff informs caregivers (family, relatives) in urgent situations	3.75	4.00	-0.619	0.172
7	Staff is sensitive to issues related to the elderly or disabled	4.14	4.00	-1.410	0.172
8	Staff arrives late at its appointments	3.87	4.00	-0.985	0.172
9	Staff informs enrollee if it is going to arrive late	4.39	5.00	-1.898	0.172
10	Improvement in social functioning via companionship	3.83	4.00	-0.619	0.172
11	Inadequate frequency of visits	2.70	3.00	0.200	0.172
12	Enrollees feel more safe and secure	4.07	4.00	-1.237	0.172
13	Staff inadequately meets certain needs of the enrollee	3.68	4.00	-0.560	0.172
14	Enrollee not obliged to ask for help of others	3.99	4.00	-1.053	0.172
15	Staff forces enrollee to do things he/she dislikes	4.23	5.00	-1.417	0.172
16	Availability of staff to listen to what the enrollee has to say by phone	3.85	4.00	-0.721	0.172
17	Enrollee avoids discussing personal issues with staff due to a lack of trust	4.23	5.00	-1.539	0.172
18	Staff takes into account enrollee's views while making decisions concerning him	3.98	4.00	-1.106	0.172
19	The program's services are of little value to the enrollee	4.14	4.00	-1.469	0.172
20	Enrollee trusts staff for providing services	4.25	4.00	-1.610	0.172
21	Staff refuses to provide some services that it should	4.24	4.00	-1.713	0.172
22	Enrolment in the program was fast	4.28	5.00	-1.528	0.172
23	Staff avoids answering questions that concern the enrollee	4.18	4.00	-1.356	0.172
24	Services provided save money by not hiring a housewife or a nurse	4.11	4.00	-1.356	0.172
25	Feeling that staff will always be available if needed by enrollee	4.09	4.00	-1.181	0.172
26	Changes are made regarding the program without asking the enrollee	4.01	4.00	-0.995	0.172
27	Staff is careful while providing services (e.g. brings back receipts from shopping)	4.23	4.00	-1.290	0.172
28	Staff causes tensions with enrollee without a reason	4.44	5.00	-2.003	0.172
29	Staff knows how to serve the enrollee properly	4.04	4.00	-1.003	0.172
30	Suitability of scheduled days and hours of visits	3.26	3.00	-0.060	0.172
31	Staff listens carefully to what the enrollee has to say	4.33	4.00	-1.610	0.172
32	Expectations from the program were higher than actual services provided	3.56	4.00	-0.585	0.172
33	Enrollee would recommend the program to others	4.41	5.00	-1.731	0.172

From the 319 home care enrollees visited, 17 were absent at the time of the visit and 91 refused to participate in the study, yielding an initial sample of 211 face-to-face interviews. Among the participants, 93 were living in Stavroupoli (44.1%), 46 in Thermaikos (21.8%), 41 in Menemeni (19.4%) and 31 in Pefka (14.7%). Six respondents were excluded failing the validity test by providing different replies in the two control questions. Four more did not respond to the majority of items. The final sample consisted of 201 usable interviews, yielding a response rate of 63% and an observation-to-item ratio of 6.48:1. There were no missing values in the remaining questionnaires. This entails clarity of the constructed items. The majority of enrollees in the final sample was women and had little or no education. Most participants were being visited 1-3 times each week by workers of the home care program and were getting additional help in their lives from their children. Their net monthly income was less than €700 (Table 2).

**Table 2:** Characteristics and descriptive statistics of the final sample

Variable	No. of Responses	%	Variable	No. of Responses	%
Level of Education			Informal Caregivers		
None	49	24.4	Relatives	25	12.4
Elementary School	117	58.2	Children	115	57.2
High School - Lyceum	26	13.0	Neighbours	29	14.4
Higher Education	2	1.0	Friends	16	8.0
University	7	3.5	Others	16	8.0
Age			Monthly Net Income (€)		
65-69	39	19.4	< 300	31	15.4
70-74	52	25.9	301 - 500	94	46.8
75-79	54	26.9	501-700	56	27.9
80-85	39	19.4	701-900	13	6.5
86-90	17	8.5	> 900	7	3.5
Gender			Family Status		
Male	75	37.3	Single	20	10.0
Female	126	62.7	Widowed	109	54.2
Visit Frequency			Divorced	21	10.4
1 per month	9	4.5	Married	51	25.4
1 per fifteen days	24	11.9	Enrolment Duration		
1 per week	90	44.8	≤ 1 year	47	23.4
2-3 per week	66	32.8	(1 year - 2 years]	80	39.8
4-5 per week	11	5.5	(2 years - 3 years]	45	22.4
Other	1	0.5	> 3 years	29	14.4

The KMO measure was 0.942 and Bartlett's test was statistically significant (p < 0.01). As can be seen in Table 3, factor analysis based on polychoric correlations and performed with unweighted least squares and varimax rotation yielded five factors, which explain 61.16% of the variance in the original 31 variables. One factor contained only one item and thus could not form a summated scale by itself. Items 2, 4, 10 and 24 made up the scale "socio-economic change", questions 3, 5, 23, 28 and 29 constituted the scale "staff skills and attitudes", items 15 and 20 the scale "service appropriateness" and questions 11 and 30 formed the scale "service planning". Note that item 20 in the "service appropriateness" scale had in fact a high factor loading also with the one-item factor mentioned above. Since that factor has been disregarded for having only one item, we decided to retain item 20 and its two-item scale, despite the fact that the difference in factor loadings was less than 0.20.

**Table 3:** Factor analysis with polychoric correlations†

Item	**1**^**st**^	**2**^**nd**^	**3**^**rd**^	**4**^**th**^	**5**^th^
1	0.485	0.326	0.324	0.190	0.190
2	**0.559**	0.273	0.335	0.148	0.037
3	0.454	**0.658**	0.180	0.078	-0.061
4	**0.656**	0.201	0.142	0.375	0.072
5	0.371	**0.688**	0.058	0.223	0.046
6	0.417	-0.079	0.553	0.128	0.227
7	0.557	0.475	0.129	0.227	0.030
8	0.278	0.307	0.186	0.048	0.361
9	0.535	0.442	0.502	0.186	0.227
10	**0677**	0.248	0.305	0.257	0.218
11	0.180	0.078	-0.011	0.018	**0.539**
12	0.597	0.327	0.446	0.265	0.132
13	0.500	0.335	0.088	0.051	0.185
14	0.550	0.265	0.199	0.426	0.207
15	0.237	0.339	**0.681**	0.129	-0.036
16	0.518	0.276	-0.019	0.558	0.096
17	0.366	0.328	0.251	0.511	0.092
18	0.260	0.263	0.249	**0.574**	0.133
19	0.435	0.116	0.244	0.455	0.321
20	0.192	0.222	**0.627**	0.545	0.073
21	0.212	0.508	0.247	0.461	0.040
22	0.324	0.545	0.183	0.358	0.363
23	0.286	**0.633**	0.109	0.250	0.284
24	**0.640**	0.242	0.229	0.231	0.135
25	0.509	0.370	0.393	0.427	0.177
26	0.093	0.469	0.354	0.124	0.352
27	0.237	0.416	0.435	0.362	0.216
28	0.244	**0.704**	0.211	0.265	0.219
29	0.162	**0.636**	0.394	0.226	0.126
30	-0.026	0.079	0.138	0.152	**0.738**
31	0.226	0.432	0.530	0.175	0.324

^† ^Factors were extracted by applying unweighted least squares with varimax rotation (with Kaiser normalization) to polychoric correlations.

The factor analysis was repeated with Pearson correlations. The variance explained was similarly high (62.97%) and - although there were some differences in the actual correlations used- the factors obtained with this approach were identical to those obtained with polychoric correlations. Hence these results are not presented here. Furthermore, given the skewed nature of the data documented in Table 1, analysis was also carried out with principal axis factoring. Results (not shown here) indicated that the factors identified were identical to those derived by unweighted least squares.

On inspection of Table 4, it is evident that Cronbach's alpha coefficients were high for the first two scales, acceptable for the "service appropriateness" scale and low for the multi-item scale that relates to service planning. The same conclusion was reached when we employed the ordinal standardised item alpha coefficient (Table 4). Note that this latter measure also implied that the overall satisfaction scale was more reliable than initially suggested. Scale data were skewed for the first three scales (skewness statistic values were -1.082, -1.921 and -1.728 with a std. error of 0.172, respectively). Only the service planning scale was found to be symmetrical (skewness statistic 0.344, st. error 0.172). We thus also examined the Spearman correlation coefficient between the items and the scale (corrected for overlap) in which factor analysis showed they belonged. Internal consistency was hence further documented for the skewed data since the non parametric correlation statistic exceeded 0.40 in all cases (Table 5). In contrast, the scale "service planning" failed this reliability criterion. In line with these findings, the values of inter-item Spearman's correlations for the first three scales ranged from 0.359 to 0.624. However, the two items we assigned to the "service planning" dimension had a correlation coefficient of only 0.280.

**Table 4:** Internal consistency reliability and satisfaction scores

Item	Scale/Item Description	Cronbach's Alpha Coefficient		Mean Satisfaction	Median Satisfaction
	SOCIO-ECONOMIC CHANGE	0.835† (0.859)‡		3.89	4.00
2	Encouragement in taking initiatives		0.813§	3.75	4.00
4	Improvement in financial condition due to the provided services		0.767	3.86	4.00
10	Improvement in social functioning via companionship		0.788	3.83	4.00
24	Services provided save money by not hiring a housewife or a nurse		0.795	4.11	4.00
	STAFF SKILLS & ATTITUDES	0.865 (0.894)		4.25	4.40
3	Staff disparages enrollee due to his/her personal problems		0.838	4.49	5.00
5	Staff is in a hurry to leave the house		0.834	4.10	4.00
23	Staff avoids answering questions that concern the enrollee		0.834	4.18	4.00
28	Staff causes tensions with enrollee without a reason		0.840	4.44	5.00
29	Staff knows how to serve the enrollee properly		0.835	4.04	4.00
	SERVICE APPROPRIATENESS	0.756 (0.777)		4.24	4.50
15	Staff forces enrollee to do things he/she dislikes		-	4.23	5.00
20	Enrollee trusts staff for providing services		-	4.25	4.00
	SERVICE PLANNING	0.520 (0.545)		2.98	3.00
11	Inadequate frequency of visits		-	2.70	3.00
30	Suitability of scheduled days and hours of visits		-	3.26	3.00
	OVERALL SATISFACTION	0.630 (0.728)		3.99	4.00
32	Expectations from the program were higher than actual services provided		-	3.56	4.00
33	Enrollee would recommend the program to others		-	4.41	5.00

† Summated scale's Cronbach coefficient.

‡ Ordinal standardised item Cronbach alpha.

§ Cronbach coefficient with specific item excluded from calculation.

**Table 5:** Multi-trait analysis with Pearson, Spearman, polychoric and polyserial correlations †

Scale/Item			Item - Scale Correlations				
Socio-Economic Change	Mean	SD	Socio-Economic Change	Staff Skills & Attitudes	Service Appropriateness	Service Planning	Scaling Success§
2	3.75	0.980	0.614‡	0.508	0.463	0.154	3
			(0.522)‡	(0.423)	(0.439)	(0.081)	
			[0.609]‡	[0.494]¶	[0.506]	[0.144]	
4	3.86	1.096	0.718‡	0.48	0.336	0.177	3
			(0.693)‡	(0.435)	(0.335)	(0.139)	
			[0.762]‡	[0.483]	[0.390]	[0.194]	
10	3.83	0.977	0.675‡	0.565	0.493	0.277	3
			(0.662)‡	(0.463)	(0.472)	(0.236)	
			[0.724]‡	[0.556]	[0.547]	[0.292]	
24	4.11	1.096	0.659‡	0.445	0.379	0.197	3
			(0.573)‡	(0.487)	(0.398)	(0.131)	
			[0.683]‡	[0.476]	[0.456]	[0.199]	
Staff Skills & Attitudes							
3	4.49	0.901	0.487	0.678‡	0.458	0.133	3
			(0.416)	(0.504)‡	(0.291)	(0.065)	
			[0.544]	[0.674]‡	[0.421]	[0.114]	
5	4.1	1.054	0.482	0.700‡	0.404	0.202	3
			(0.463)	(0.609)‡	(0.321)	(0.140)	
			[0.519]	[0.688] ‡	[0.403]	[0.195]	
23	4.18	0.917	0.486	0.695‡	0.415	0.325	3
			(0.397)	(0.615)‡	(0.287)	(0.275)	
			[0.497]	[0.709]‡	[0.386]	[0.352]	
28	4.44	0.893	0.496	0.671‡	0.500	0.261	3
			(0.424)	(0.601)‡	(0.327)	(0.147)	
			[0.539]	[0.726]‡	[0.453]	[0.257]	
29	4.04	0.940	0.500	0.690‡	0.536	0.195	3
			(0.414)	(0.570)‡	(0.498)	(0.130)	
			[0.503]	[0.687]‡	[0.579]	[0.199]	
Service Appropriateness							
15	4.23	1.076	0.414	0.495	0.612‡	0.060	3
			(0.390)	(0.354)	(0.447)‡	(0.018)	
			[0.448]	[0.472]	[0.636]‡	[0.050]	
20	4.25	0.944	0.501	0.519	0.612‡	0.229	3
			(0.434)	(0.395)	(0.447)‡	(0.132)	
			[0.522]	[0.494]	[0.636]‡	[0.227]	
Service Planning							
11	2.7	1.036	0.199	0.202	0.076	0.353‡	3
			(0.156)	(0.122)	(0.024)	(0.280)‡	
			[0.219]	[0.219]	[0.052]	[0.375]‡	
30	3.26	0.930	0.204	0.256	0.185	0.353‡	3
			(0.102)	(0.163)	(0.112)	(0.280)‡	
			[0.209]	[0.275]	[0.163]	[0.375]‡	

† Spearman correlation coefficients are shown in parentheses. ‡ Item-total correlation corrected for overlap. § Number of correlation comparisons in which the item-scale correlation was found to be significantly higher for hypothesized scale than for competing scale. Significance level was set at 5%. In brackets polychoric or polyserial correlations were computed, according to whether one of the two variables to be correlated had more than 15 categories and could thus be treated as continuous.

Table 6 reports test-retest correlations. 52 enrollees were approached and 36 had agreed to complete the questionnaire for a second time, a response rate of 69.23%. The values of the statistics are considered to be satisfactory for all scales.

**Table 6:** Test-retest reliability coefficients

Summated Scale	Intraclass Coefficient†	95% CI of Intraclass Correlation		Polychoric Correlation	Kendall's Tau b Coefficient
		**Minimum**	**Maximum**		

Socio-economic Change	0.958	0.919	0.978	0.942**	0.835**
Staff Skills & Attitudes	0.926	0.858	0.962	0.949**	0.830**
Service Appropriateness	0.815	0.669	0.901	0.894**	0.734**
Service Planning	0.946	0.898	0.972	0.982**	0.902**
Overall Satisfaction	0.891	0.799	0.943	0.936**	0.827**

† Model ICC (2,1) of SPSS v.15.0 has been used (i.e. single measure two-way random effects model for absolute agreement, see [50]).

** p < 0.01 (2-tailed).

Multitrait scaling yielded satisfactory results. Table 5 shows that the first criterion of item internal consistency was met for three scales, since the Pearson, Spearman and polychoric correlations took values above 0.40. It was not satisfied, however, for the service planning scale. The second criterion of divergent validity was fully met for all scales. Regardless of which correlation coefficient we employed, in all cases the correlation of an item with its own scale was higher than its correlation with other summated scales. Differences were in fact significant (p < 0.05). With respect to the third criterion, for the two scales that contained more than two items, the ranges of the various correlation coefficient values were quite narrow. Similar values were also evident for the variances in the four summated scales, suggesting that the last criterion of the multitrait analysis was also satisfied.

Inter-scale correlations are reported in Table 7. For the scales "socio-economic change", "staff skills & attitudes" and "service appropriateness" spearman, polychoric and polyserial correlations were all higher than the suggested literature value of 0.40 and significant (p < 0.05), whereas Kendall Tau coefficients in some cases fell a little short of this and yet were still statistically significant. Furthermore, as expected, summated scale scores were positively related to overall satisfaction. Regarding "service planning", the criterion for the inter-scale correlations was not met and the relationship with overall satisfaction was weaker than that of other scales with the same global measure.

**Table 7:** Correlations between scale scores and respondents' characteristics †

Scale/Characteristic	Socio-Economic Change	Staff Skills & Attitudes	Service Appropriateness	Service Planning
Overall Satisfaction Scale	0.689***	0.653***	0.402***	0.336**
	(0.570)***	(0.536)***	(0.337)***	(0.269)**
	[0.472]***	[0.806]***	[0.434]***	[0.391]**
Socio-Economic Change	-	0.542***	0.491***	0.158**
	-	(0.426)***	(0.388)***	(0.123)**
	-	[0.675]***	[0.480]***	[0.261]***
Staff Skills & Attitudes	0.542***	-	0.425***	0.176**
	(0.426)***	-	(0.347)***	(0.139)**
	[0.675]***	-	[0.518]***	[0.296]***
Service Appropriateness	0.491***	0.425***	-	0.058
	(0.388)***	(0.347)***	-	-0.049
	[0.480]***	[0.518]***	-	[0.115]**
Service Planning	0.158**	0.176**	0.058	-
	(0.123)**	(0.139)**	-0.049	-
	[0.261]***	[0.296]***	[0.115]**	-
Age	0.005	-0.060	0.076	0.127
	(0.004)	(-0.048)	(0.060)	-0.103
	[0.045]	[-0.073]	[0.054]	[0.160]
Education	-0.103	-0.024	-0.105	-0.130
	(-0.078)	(-0.019)	(-0.090)	(-0.110)
	[-0.132]	[-0.077]	[-0.122]	[-0.143]
Visit Frequency	0.026	0.076	-0.019	0.033
	(0.019)	(0.063)	(-0.013)	(0.026)
	[0.060]	[0.107]	[0.006]	[0.043]**
Income	-0.215)***	-0.150**	-0.157**	-0.130
	(-0.169)***	(-0.123)**	(-0.126)**	(-0.108)
	[-0.203]*	[-0.091]*	[-0.127]*	[-0.180]
Enrolment Duration	0.090	0.026	0.183***	0.114
	(0.068)	(0.022)	(0.142)**	(0.087)
	[0.074]	[0.043]	[0.177]	[0.096]*

† Data outside parentheses are Spearman's correlations, whereas figures inside them represent Kendall's Tau b correlations. Polychoric or polyserial correlations are reported inside brackets.

*** p < 0.01 (2-tailed).

** p < 0.05 (2-tailed).

* p < 0.10 (2-tailed).

Finally, Spearman's and Kendall's Tau correlations suggest some significant relationships of scales with respondents' characteristics that conform to our prior beliefs. As expected, net monthly income was negatively related to satisfaction with socio-economic change, staff skills & attitudes and service appropriateness. It was unrelated to service planning satisfaction. In addition, the duration of enrolment might be positively related to satisfaction with service appropriateness. These results however should be interpreted with some caution since polychoric and polyserial correlations lacked statistical significance (p > 0.05). These latter correlations also revealed an expected significant positive relation between visit frequency and satisfaction with service planning.

The percentages of observations falling into the highest satisfaction score category for the four summated scales were 7%, 15.4%, 35.3% and 3.5%, respectively. The respective floor effects were very limited and equal to 0.5%, 0.5%, 2% and 2.5%. Note that high ceiling effects are not uncommon in the literature [58], although some studies do find such effects to be more limited, that is lower than 10-12% [59].

Median (mean) satisfaction from socio-economic aspects of care, staff skills and attitudes, appropriateness of provided services and planning was 75.00 (72.17), 85.00 (81.27), 87.5 (81.03) and 60.00 (59.60), respectively. Overall satisfaction was 75.00 (74.63). This means that most enrollees were satisfied yet not very satisfied overall with provided services. In fact, 81.6% of all participants were to some extent satisfied with the home care programs, whilst 9% expressed some level of dissatisfaction. A point of concern, nevertheless, relates to the lower satisfaction with service planning documented.

## Discussion

A preliminary validation of a new psychometric tool for measuring satisfaction with home care was conducted with a sample of 201 individuals enrolled in five home care programs operating in the City of Thessaloniki. The questionnaire had 31 items and was expressed on a Likert scale. Although this scale has been typically considered in practice as interval it is in principle ordinal. We have therefore extended the usual research design to comparatively include ordinal methods, such as polychoric correlations.

Results from this exploratory analysis suggested that four multi-item factors are associated with home care satisfaction: socioeconomic change (4 items), staff skills and attitudes (5 items), appropriateness of provided services (2 items), and planning of services (2 items). An additional two-item scale was formed reflecting overall satisfaction with the programs. The first three multi-item scales had satisfactory internal consistency reliability, test-retest reliability and construct validity. An additional dimension suggested by factor analysis was "service planning" which has been found to have lower internal consistency reliability. Future research should further investigate whether the specific summated scale can be improved or legitimately ignored without sacrificing content validity.

There are differences as well as similarities between our scale dimensions and those found in other international studies. For instance, Reeder & Chen's confirmatory factor analysis lumped together three previously documented scales -namely professional/technical, interpersonal/trust and educational- into one unique dimension [13]. Using the same instrument, Laferriere found four dimensions of client satisfaction with home nursing care: technical quality of care, communication, personal relationships between client and provider, and service delivery [11]. Bear *et al*. instead reported two factors: service delivery and service sufficiency [20]. Netten *et al*. and Jones *et al*. mentioned carer quality - opinions towards carer, service quality and outcomes [27,60]. Finally, Geron *et al*. classified items into the categories homemaker/health aide, care management service, home-delivered meal service and grocery service [22]. The reasons for the observed differences between the number and content of scales in the home care satisfaction literature have not been studied. In our research some dissimilarity with other findings was expected due to the differences in the Greek home care service provision outlined previously.

There is some evidence to suggest that satisfaction ratings with home care are influenced by monthly income and duration of enrolment in the program. Individuals with fewer own financial resources were in most need of home care services and hence reported higher satisfaction, and vice versa. Further, the more years an enrollee had been participating in the home care program, the more he trusted staff to carry out tasks for him and presumably did not allow aides to force him do things he disliked. Yet one should be cautious in uncritically adopting these associations and explanations since some correlation measures lacked statistical significance.

It is important to note that the results of this study were not influenced by the approach employed. That is, whether one assumed that the 5-point Likert scale was an interval scale or an ordinal one had in essence no significant effect in the study findings. Similar findings from ordinal and Pearsonian exploratory factor analyses have also been documented for the Big Five Questionnaire [39].

Finally, overall enrollees were satisfied with the home care programs. Higher levels of satisfaction were associated with the skills and attitudes of the staff and the suitability of services. Whereas lower levels were associated with the social and economic aspects of provided help and service planning. There are no existing studies in the Greek setting with which to compare the current findings. Although home care services in other countries differ, have been evaluated with other tools and refer to different populations, we simply note that findings such as ours are not uncommon. For instance, the mean satisfaction in this study was 74.63, whereas in a US study a mean of 74.1 was reported [22].

This study has certain limitations related both to the measurement of satisfaction and the validation of the questionnaire. Since the provision of home care programs in Greece is decentralized and information on the characteristics of the population of enrollees is not gathered by the central government, we were unable to investigate whether our sample was representative. The sample was in fact geographically restricted to the city of Thessaloniki. Moreover, there were no other studies in the Greek setting with which we could compare our findings. The summated scales "service planning" and "overall satisfaction" had low reliability values and "service appropriateness" a marginally acceptable Cronbach's alpha coefficient. The fact that these were all two-item scales might partly account for that. Nevertheless, we note that scales comprising of only two questions have often been found highly reliable in the Greek setting [4]. Finally, the measurement of satisfaction has been criticized for its weak conceptual foundation and potential biases due to acquiescent response set [61,62].

## Conclusions

Summing up, exploratory ordinal and traditional factor analyses alongside reliability and validity tests revealed three reliable and valid multi-item scales: socioeconomic change, staff skills and attitudes and appropriateness of services. A fourth dimension -service planning- had lower internal consistency reliability. Overall, enrollees were satisfied with the home care program. Future research should further validate the questionnaire with the use of confirmatory factor analysis and larger samples from other home care programs. Moreover, the role of service planning in the determination of home care satisfaction should be further assessed.

## Competing interests

The authors declare that they have no competing interests.

## Authors' contributions

VHA conceived and designed the study, helped to finalize the tool, carried out data analysis and interpretation, and drafted the manuscript. AK and MT reviewed the literature, drafted the questionnaire and carried out the study administration. DN designed and supervised the study. AN contributed to all the statistical analyses. All authors read and approved the final manuscript.

## Pre-publication history

The pre-publication history for this paper can be accessed here:

http://www.biomedcentral.com/1472-6963/10/189/prepub

## Supplementary Material

Additional file 1 **Home Care Satisfaction Questionnaire**. A questionnaire in Greek that measures satisfaction with home care services. Click here for file
