# Supplementary material for: Development and preliminary validation of a questionnaire to measure satisfaction with home care in Greece: an exploratory factor analysis of polychoric correlations
Source: BMC Health Serv Res. 2010 Jul 5;10:189. doi: 10.1186/1472-6963-10-189 (PMC2912895; doi:10.1186/1472-6963-10-189)
Supplement: Additional file 1 — Home Care Satisfaction Questionnaire. A questionnaire in Greek that measures satisfaction with home care services. [file 1472-6963-10-189-S1.PDF]

ΕΡΩΤΗΜΑΤΟΛΟΓΙΟ ΙΚΑΝΟΠΟΙΗΣΗΣ
ΑΠΟ ΤΟ ΠΡΟΓΡΑΜΜΑ «ΒΟΗΘΕΙΑ ΣΤΟ ΣΠΙΤΙ»

Α/Α	Ε Ρ Ω Τ Η Σ Η	1<br>ΔΙΑΦΩΝΩ ΑΠΟΛΥΤΑ	2<br>ΔΙΑΦΩΝΩ	3<br>ΟΥΤΕ ΣΥΜΦΩΝΩ ΟΥΤΕ ΔΙΑΦΩΝΩ	4<br>ΣΥΜΦΩΝΩ	5<br>ΣΥΜΦΩΝΩ ΑΠΟΛΥΤΑ
**1**	Το προσωπικό με βοηθάει να ξεπερνάω τα προβλήματα που αντιμετωπίζω όσο είναι δυνατό.					
**2**	Θεωρώ ότι το προσωπικό χρειάζεται εκπαίδευση. (Ερώτηση Ελέγχου)					
**3**	Το προσωπικό με βοηθάει να παίρνω πρωτοβουλίες για θέματα που με αφορούν					
**4**	Αισθάνομαι ότι το προσωπικό με βλέπει υποτιμητικά εξαιτίας των προβλημάτων που αντιμετωπίζω.					
**5**	Η εγγραφή μου στο "Βοήθεια στο Σπίτι" έχει βοηθήσει στη βελτίωση της οικονομικής μου κατάστασης (π.χ. μεσολάβηση για την έκδοση επιδομάτων, παροχή ιατροφαρμακευτικού υλικού, οικιακές εργασίες).					
**6**	Αισθάνομαι ότι το προσωπικό βιάζεται να φύγει από το σπίτι μου.					
**7**	Το προσωπικό ενημερώνει την οικογένεια μου ή το συγγενικό μου περιβάλλον όταν υπάρχει ανάγκη					
**8**	Θεωρώ ότι έπρεπε να ασχολείται μεγαλύτερος αριθμός προσωπικού (π.χ, νοσοκόμες, οικογενειακοί βοηθοί, κοινωνικοί λειτουργοί).					
**9**	Το προσωπικό είναι ευαισθητοποιημένο σε θέματα που αφορούν τους ηλικιωμένους.					
**10**	Το προσωπικό καθυστερεί να έρθει στο ραντεβού στο σπίτι μου.					
**11**	Το προσωπικό πάντοτε με ενημερώνει τηλεφωνικά όταν πρόκειται να καθυστερήσει να έρθει στο σπίτι μου.					
**12**	Αισθάνομαι ότι το προσωπικό αδιαφορεί για τα προβλήματα μου (Ερώτηση Ελέγχου)					

ΚΩΣΤΑΡΕΛΗΣ Α., ΤΣΙΤΟΥΡΙΔΟΥ Μ., ΑΛΕΤΡΑΣ Β., ΝΙΑΚΑΣ Δ. © 2006

Α/Α	ΕΡΩΤΗΣΗ	1 ΔΙΑΦΩΝΩ ΑΠΟΛΥΤΑ	2 ΔΙΑΦΩΝΩ	3 ΟΥΤΕ ΣΥΜΦΩΝΩ ΟΥΤΕ ΔΙΑΦΩΝΩ	4 ΣΥΜΦΩΝΩ	5 ΣΥΜΦΩΝΩ ΑΠΟΛΥΤΑ
**13**	Νιώθω ότι έχει βελτιωθεί η κοινωνική μου ζωή (έχω τακτικά συντροφιά) μετά την εγγραφή μου στο "Βοήθεια στο Σπίτι".					
**14**	Νομίζω ότι το προσωπικό πρέπει να με επισκέπτεται περισσότερες φορές (μέρες).					
**15**	Έχω ένα αίσθημα ασφάλειας από τότε που γράφτηκα στο "Βοήθεια στο Σπίτι" επειδή το προσωπικό είναι έμπειρο.					
**16**	Νομίζω ότι το προσωπικό καλύπτει ανεπαρκώς ορισμένες μου ανάγκες.					
**17**	Η εγγραφή μου στο "Βοήθεια στο Σπίτι" συνέβαλε ώστε να σταματήσω να υποχρεώνομαι στους άλλους (για τα ψώνια, την αγορά και την εγγραφή φαρμάκων, για τη μέτρηση της πίεσης).					
**18**	Το προσωπικό με πιέζει να κάνω πράγματα που με δυσαρεστούν (π.χ. να συζητώ θέματα που είναι προσωπικά μου, μου απαγορεύει να τρώω φαγητά που με αρέσουν).					
**19**	Όταν τηλεφωνώ στην υπηρεσία υπάρχει πάντοτε κάποιο άτομο για να μιλήσω μαζί του και να του πω αυτό που θέλω.					
**20**	Αποφεύγω να μιλάω για προσωπικά μου θέματα στο προσωπικό επειδή υπάρχει έλλειψη εμπιστοσύνης από εμένα για το προσωπικό.					
**21**	Το προσωπικό πάντοτε λαμβάνει υπόψη του τη γνώμη μου στις περιπτώσεις λήψης αποφάσεων που με αφορούν.					
**22**	Οι υπηρεσίες που μου προσφέρει το "Βοήθεια στο Σπίτι" είναι μικρής σημασίας για εμένα.					
**23**	Έχω εμπιστοσύνη στο προσωπικό όταν του ζητάω να μου κάνει μια εξυπηρέτηση.					
**24**	Το προσωπικό αρνείται να με εξυπηρετεί όταν του ζητάω εργασίες οι οποίες όμως αποτελούν μέρος των καθηκόντων του.					
**25**	Η εγγραφή μου στο "Βοήθεια στο Σπίτι" έγινε σε σύντομο χρονικό διάστημα.					

ΚΩΣΤΑΡΕΛΗΣ Α., ΤΣΙΤΟΥΡΙΔΟΥ Μ., ΑΛΕΤΡΑΣ Β., ΝΙΑΚΑΣ Δ. © 2006

Α/Α	ΕΡΩΤΗΣΗ	1 ΔΙΑΦΩΝΩ ΑΠΟΛΥΤΑ	2 ΔΙΑΦΩΝΩ	3 ΟΥΤΕ ΣΥΜΦΩΝΩ ΟΥΤΕ ΔΙΑΦΩΝΩ	4 ΣΥΜΦΩΝΩ	5 ΣΥΜΦΩΝΩ ΑΠΟΛΥΤΑ
**26**	Το προσωπικό αποφεύγει να μου απαντήσει σε ερωτήσεις που κάνω για θέματα που με αφορούν.					
**27**	Το "Βοήθεια στο Σπίτι" μου προσφέρει υπηρεσίες οι οποίες θα με επιβάρυναν οικονομικά (θα έπρεπε να πληρώνω οικιακή βοηθό ή νοσοκόμα στο σπίτι).					
**28**	Η υπηρεσία "Βοήθεια στο Σπίτι" έχει ανάγκη από προσωπικό και άλλων ειδικοτήτων (π.χ. γιατρό, ή φυσιοθεραπευτή ή κάτι άλλο).					
**29**	Νιώθω ότι άμα έχω κάποια ανάγκη πάντοτε θα υπάρχει κάποιος από το προσωπικό κοντά μου.					
**30**	Το προσωπικό του "Βοήθεια στο Σπίτι" κάνει αλλαγές στο πρόγραμμα χωρίς να ερωτηθώ.					
**31**	Το προσωπικό είναι πολύ προσεκτικό όταν κάνει δουλειές για μένα (π.χ. όταν κάνει οικιακές εργασίες, μου φέρνει τις αποδείξεις από τα ψώνια).					
**32**	Πιστεύω ότι υπάρχουν φορές που το προσωπικό συγκρούεται μαζί μου χωρίς να έχω δώσει κάποια αφορμή.					
**33**	Το προσωπικό γνωρίζει πώς να με εξυπηρετεί (να κάνει εργασίες για μένα) όταν του το ζητάω.					
**34**	Θεωρώ ότι το προσωπικό πρέπει να έρχεται άλλες ώρες ή και μέρες από αυτές που έρχεται στο σπίτι μου.					
**35**	Το προσωπικό με ακούει προσεκτικά όταν συζητάμε (αποφεύγει να με διακόπτει, έχει υπομονή όταν με ακούει).					
**36**	Οι προσδοκίες που είχα όταν γράφτηκα στο πρόγραμμα "Βοήθεια στο Σπίτι" ήταν υψηλότερες σε σχέση με τις υπηρεσίες που μου προσφέρει.					
**37**	Θα πρότεινα και σε άλλα άτομα να εγγραφούν στην υπηρεσία "Βοήθεια στο Σπίτι".					

ΚΩΣΤΑΡΕΛΗΣ Α., ΤΣΙΤΟΥΡΙΔΟΥ Μ., ΑΛΕΤΡΑΣ Β., ΝΙΑΚΑΣ Δ. © 2006

ΔΗΜΟΓΡΑΦΙΚΑ ΣΤΟΙΧΕΙΑ ΕΞΥΠΗΡΕΤΟΥΜΕΝΟΥ

1. Φύλο

Άνδρας	
Γυναίκα	

2. Ηλικία

65-69	70-74	75-79	80-85	86-90

3. Μόρφωση

Αναλφάβητος/η	Δημοτικό	Γυμνάσιο	Λύκειο	ΤΕΙ	ΑΕΙ	Άλλο

4. Οικογενειακή Κατάσταση

Άγαμος/η	Παντρεμένος/η	Χήρος/α	Διαζυγευμένος/η

5. Μηνιαίο Εισόδημα (σε Ευρώ)

Έως 300 €	301 € - 500 €	501 € - 700 €	701 € - 900 €	901 € και πάνω

6. Περίοδος συμμετοχής στο Πρόγραμμα Βοήθεια στο Σπίτι

2-6 μήνες	7-12 μήνες	13-18 μήνες	19-24 μήνες	25-30 μήνες	31-36 μήνες	36 μήνες και πάνω

7. Συχνότητα επισκέψεων

1 φορά την εβδομάδα	2-3 φορές την εβδομάδα	4-5 φορές την εβδομάδα	1 φορά στις 15 ημέρες	1 φορά τον μήνα	Άλλο

8. Εκτός από το Πρόγραμμα Βοήθεια στο Σπίτι, βοήθεια σας προσφέρουν

Συγγενείς	Παιδιά	Γείτονες	Φίλοι	Άλλο

ΚΩΣΤΑΡΕΛΗΣ Α., ΤΣΙΤΟΥΡΙΔΟΥ Μ., ΑΛΕΤΡΑΣ Β., ΝΙΑΚΑΣ Δ. © 2006
